# Rolling Bearing Fault Diagnosis Based on Optimal Notch Filter and Enhanced Singular Value Decomposition

**DOI:** 10.3390/e20070482

**Published:** 2018-06-21

**Authors:** Bin Pang, Yuling He, Guiji Tang, Chong Zhou, Tian Tian

**Affiliations:** School of Energy, Power and Mechanical Engineering, North China Electric Power University, Baoding 071000, China

**Keywords:** Teager energy entropy, notch filter, enhanced singular value decomposition, rolling bearings, fault diagnosis

## Abstract

The impulsive fault feature signal of rolling bearings at the early failure stage is easily contaminated by the fundamental frequency (i.e., the rotation frequency of the shaft) signal and background noise. To address this problem, this paper puts forward a rolling bearing weak fault diagnosis method with the combination of optimal notch filter and enhanced singular value decomposition. Firstly, in order to eliminate the interference of the fundamental frequency signal, the original signal was processed by the notch filter with the fundamental frequency as the center frequency and with a varying bandwidth to get a series of corresponding notch filter signals. Secondly, the Teager energy entropy index was adopted to adaptively determine the optimal bandwidth to complete the optimal notch filter analysis on the raw vibration signal and obtain the corresponding optimal notch filter signal. Thirdly, an enhanced singular value decomposition de-nosing method was employed to de-noise the optimal notch filter signal. Finally, the envelope spectrum analysis was conducted on the de-noised signal to extract the fault characteristic frequencies. The effectiveness of the presented method was demonstrated via simulation and experiment verifications. In addition, the minimum entropy deconvolution, Kurtogram and Infogram methods were employed for comparisons to show the advantages of the presented method.

## 1. Introduction

Rolling bearings are one of the most critical, but one of the most easily damaged parts of rotating machinery [1]. If the defects of bearings can not be detected in time, it will cause severe safety accidents [2]. Developing effective techniques to diagnose the faults of rolling bearings as early as possible is significant in preventing the adverse effects caused by the deterioration of bearing faults [3].

The actual vibration signals of bearings with defects consist of multiple components, and the impulsive fault feature signals are easily affected by the background noise, harmonic interference signals and transmission path [4]. Therefore, plenty of signal processing methods, such as de-noising analysis, deconvolution, signal decomposition and resonance demodulation, have been introduced to address the issue of early fault diagnosis of bearings under different situations. To reduce the effect of noise on the fault feature signals, different kinds of signal de-noising methods such as singular value decomposition (SVD) [5], mathematical morphology filter [6], nonlocal means de-noising [7], and stochastic resonance [8] have been widely used to de-noise the vibration signal of bearings. Although these developed de-noising methods can pure the vibration signal, they can’t remove the other interference signals except background noise. It is still very difficult to identify the fault features when other interference signals except background noise exist. Therefore, the signal de-noising approaches are always combined with some other signal analysis approaches to get a good fault diagnosis result [9]. Deconvolution approaches such as minimum entropy deconvolution (MED) [10], maximum correlated kurtosis deconvolution (MCKD) [11], sparse maximum harmonics-to-noise-ratio deconvolution (SMHD) [12], Lucy-Richardson deconvolution [13] and multipoint optimal minimum entropy deconvolution [14] have been researched and widely used to recover the impulsive fault feature signal from the measured vibration signal. Some indicators, such as kurtosis, maximum correlated kurtosis and harmonics-to-noise-ratio are taken as the targets and a designed FIR filter is performed on the vibration signal to obtain the filtered output reaches a maximum value of the target [15,16]. The deconvolution methods have shown their effectiveness in detecting bearing faults, but they have some limitations. MED takes the kurtosis indicator as the target. Kurtosis is sensitive to random impulses, which can cause erroneous deconvolution. For MCKD, complex parameter problems must be solved in order to achieve superior results. In the principle of SMHD, the harmonics-to-noise-ratio index should be accurately estimated by using prior knowledge. Lucy-Richardson deconvolution seems to be ineffective under the condition of strong background noise. To separate the fault feature signal from the interferences, various self adaptive decomposition techniques, such as empirical mode decomposition [17], variational mode decomposition [18], and empirical wavelet decomposition [19] have been adopted for bearing failure detection. The fault features may be carried by one of the decomposed component signals, but the abnormal events, including noise and other types of interferences, can cause the problem of mode mixing. When mode mixing occurs, the fault characteristics are difficult to identify [20]. Resonance demodulation methods such as Kurtogram [21], Autogram [22], and Infogram [23] have been widely employed to detect transient vibration signatures. The analysis results of the resonance demodulation methods are mainly affected by two aspects: (a) the segmentation of the frequency bands; (b) the selection indicator of the optimal frequency band. If the fault feature signal can’t be divided into one of the frequency bands successfully or the optimal frequency band is errousneously selected, the resonance demodulation methods will lose efficiency [24,25].

As a supporting component, the rolling bearing contacts with the rotation shaft directly so that the fundamental frequency signal, i.e., the rotating frequency signal, performs very prominent in the acquired vibration signals. The fundamental frequency signal is one of the most common harmonic interference signals. In addition, the background noise seems to be always an influence factor. With the compound influence of the fundamental frequency and the noise, the impulsive fault feature information tends to be easily submerged and difficult to be recognized and traditional bearing fault diagnosis methods lack pertinence for this fault situation. It is of great significance to develop an effective technique to eliminate the compound interference of the fundamental frequency signal and background noise. Notch filter with a designed center frequency and bandwidth can directly suppress the harmonic components of the signal [26]. Considering the excellent characteristics of notch filter in restraining the harmonics, the notch filter analysis is adopted to inhibit the interference of the fundamental frequency signal by setting the fundamental frequency as the center frequency in this paper. Compared with the de-noising, deconvolution, signal decomposition and resonance demodulation methods, the notch filter is more targeted for suppressing the fundamental frequency signal. Previous studies demonstrate that the bandwidth of notch filter is a key parameter that can directly impact the analysis results [27]. To set the optimal bandwidth of the notch filter adaptively, it is necessary to find an effective index to measure the richness of the fault feature information contained in the notch filter signals corresponding to different bandwidths. Teager energy operator (TEO) has the function of tracking and strengthening the impulsive shocks [28]. Entropy is able to reflect the regularity of the impulsive shocks [29]. Combing the advantages of TEO and entropy, Teager energy entropy (TEE), as an impulsive characteristic evaluation index, is presented in this paper to determine the optimal bandwidth of the notch filter adaptively. The filtered signal corresponding to the optimal bandwidth is called optimal notch filter signal in this paper. Singular value decomposition (SVD) is an effective approach for signal de-noising, which has been widely used in bearing fault diagnosis due to its superiority over other de-noising methods [30]. Based on the principle of SVD, we proposed the enhanced singular value decomposition (ESVD) method to suppress the noise carried by the optimal notch filter signal more effectively. A novel bearing fault diagnosis method with the combination of optimal notch filter based on TEE index and ESVD de-noising was presented to eliminate the compound interference of the fundamental frequency signal and background noise. The validity of the proposed method was verified by simulated and experimental signals.

The remained contents are organized as follows: Section 2 gives a specific introduction of the theory background, the principle of the notch filter is presented, a TEE index is introduced and evaluated, detailed steps of the presented method are exhibited. The presented method is investigated by simulated and experimental signals in Section 3 and Section 4 individually. Finally, Section 5 presents a few conclusions.

## 2. Theory Background

### 2.1. Optimal Notch Filter Based on Teager Energy Entropy Index

#### 2.1.1. Notch Filter

Notch filter is an effective tool to eliminate the harmonic interferences. The basic principle of notch filter to eliminate the harmonic interferences is conducting a narrow bandwidth filter on the original. By setting the center frequency of the notch filter as the rotation frequency of the shaft, the fundamental frequency signal can be inhibited. So, the notch filter is adopted in this paper for processing the vibration signal of bearings.

The second order digital notch filter is discussed in this part. To filter the harmonic interference signal with the frequency of *ω*_0_, the frequency- amplitude curve of the filter should be zero at *ω*_0_ and almost be constant at the other frequencies [26].

When the zero point satisfies, $z=e^{\pm j\omega_{0}}$, the frequency-amplitude curve of the filter is zero at the frequencies of *ω* = ±*ω*_0_. In order to increase the amplitude to a constant instantly once the frequencies are not ±*ω*_0_, two extreme points are set near the zero points. The extreme point is described as: $z=re^{\pm j\omega_{0}}$, and the transfer function of the notch filter is shown in Equation (1):

(1) $$H(z)=\frac{b_{0}(z-e^{j\omega_{0}})(z-e^{j\omega_{0}})}{\left(z-re^{j\omega_{0}})(z-re^{j\omega_{0}}\right)}=\frac{b_{0}[1-(2\cos\omega_{0})z^{-1}+z^{-2}]}{1-2r(\cos\omega_{0})z^{-1}+r^{2}z^{-2}}$$

where 0 ≤ *r *≤ 1, the value of which depends on the bandwidth of the notch filter, *b*_0 _denotes the gain factor. The frequency band of notch filter at −3 dB is defined as the bandwidth (*Bw*). The bandwidth *Bw* and the gain coefficient *b*_0_ can be expressed as:

(2) $$Bw=(1-r)f_{s},\;\;b_{0}=\frac{\left|1-2r\cos\omega_{0}+r^{2}\right|}{2\left|1-\cos\omega_{0}\right|}.$$

where *f_s_*represents the sampling frequency. When normalized frequency is used, the sampling frequency *f_s_* is 1 and the bandwidth *Bw* equals to 1 − *r*. The frequency-amplitude curve of notch filter varies with *Bw*.

**Figure 1:**
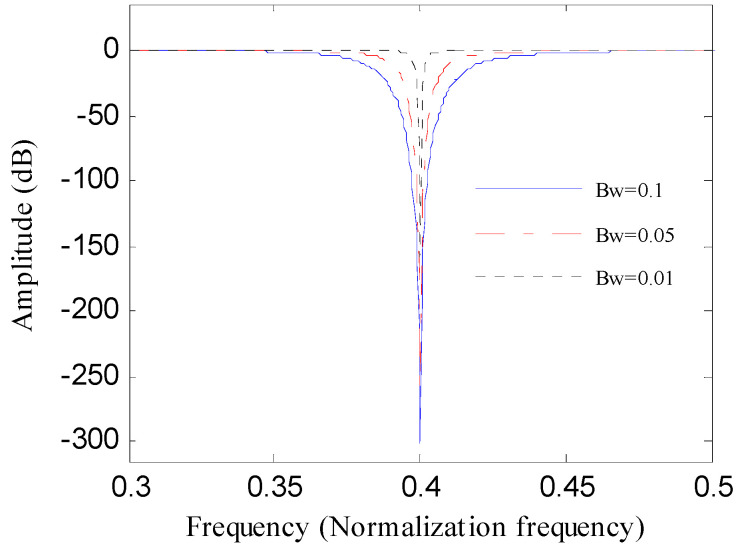
Frequency-amplitude curve of the notch with different bandwidths.

Figure 1 displays the frequency-amplitude curve of the notch filter with the bandwidth of 0.01, 0.05 and 0.1, respectively. As depicted in Figure 1, the amplitude of the frequency components around *ω *= ±*ω*_0_ varies severely when *Bw *changes. Once *Bw *decreases, the depth of notch will decrease as well. The frequency-amplitude curve of the notch filter demonstrates that the selection of *Bw* has a remarkable influence on the analysis results of notch filter. A proper value of *Bw *should be selected to achieve the best result when applying the notch filter to the vibration signals of bearings.

#### 2.1.2. Teager Energy Entropy Index

When local defects appear in bearings, periodic impulses will be generated. Some fault diagnosis methods of bearings are parametric-based analysis methods. In order to get the optimal results, a number of evaluation indexes have been employed by scholars to adaptively choose the parameters. Kurtosis, as a fourth-order cumulative statistic, is one of the most well known and frequently used indexes. A higher value of the kurtosis index represents the periodic impulsive features of the signal are richer. But kurtosis is very sensitive to the accidental impulses [31]. When an accidental impulse appears, the kurtosis value will increase sharply, which may lead to erroneous evaluations. In order to overcome the shortcoming of the kurtosis index, a novel evaluation index, called Teager energy entropy (TEE), which combines the advantages of Teager energy operator (TEO) and Shannon entropy, is introduced to select the optimal *Bw* of the notch filter to implement the optimal notch filter analysis.

The TEO of one dimensional signal *s*(*t*) has the expression as [28]:

(3) $$\Psi [s(t)]={\left[\dot{s}(t)\right]}^{2}-s(t)\ddot{s}(t).$$

And Equation (4) shows the corresponding discrete form:

(4) $$\Psi [s(n)]={\left[s(n)\right]}^{2}-s(n+1)s(n-1).$$

As reported in [28], TEO has a good quality of tracking the instantaneous energy of the signal, and this quality has been widely used for enhancing the impulsive features of the vibration signal.

The entropy is able to reflect the sparsity of the signal and has shown its potentiality for fault diagnosis [32,33]. By calculating the Shannon entropy [34] of the instantaneous energy signal obtained by performing TEO on the signal, a new index, that is the TEE index, can be described by Equation (5):

(5) $$\{\begin{array}{c} TEE=-\sum_{j=1}^{N}p_{j}\ln p_{j}\\ p_{j}=Q(j)/\sum_{j=1}^{N}Q(j),Q(j)=abs(\Psi [s(j)]) \end{array}.$$

When the original signal carries more impulse shocks, the sparsity of the signal is higher and the TEE value is smaller. In this paper, the TEE index is used to evaluate the impulsive features of the notch filtered signals to determine the optimal bandwidth and the corresponding optimal notch filter signal.

To validate the effectiveness of the TEE index in reflecting the richness of the periodic impulsive shocks of the signal and the advantage of the TEE index over the kurtosis index in overcoming the influence of the accidental impulses, five simulated signals were designed with the sampling frequency of 8192 Hz for evaluation.

The first simulated signal *x*_1_(*t*) is used to simulate the periodic impulse signal of the outer race fault, which is shown in Equation (6) [35]:

(6) $$x_{1}(t)=[\sum_{i=0}^{M-1}Dh(t-iT_{o})]*[Ae^{-\xi 2\pi f_{n}t}\cos(2\pi f_{d}t-\theta )],$$

where *M *denotes the number of the impulsive pulses, *D* is the pulse amplitude, *h*(*t*) indicates the unit pulse function, *T_o_* is the interval of the impulsive pulses, *ξ* is the structural damping factor, *f_n _*represents the natural frequency of the system, $f_{d}=f_{n}\sqrt{1-\xi^{2}}$ is the damping natural frequency, *A* and *θ *represent the amplitude and initial phase respectively.

The parameters were set as: *M *= 25, *D *= 1, *T_o _*= 0.01 s, *A *= 2, *ξ *= 0.06, *f_n _*= 2000 Hz and *θ *= 0.

Equation (7) is the expression of the second simulated signal *x*_2_(*t*), which is used to simulate the fundamental frequency signal:

(7) $$x_{2}(t)=6\sin(2\pi f_{1}t),$$

where *f*_1_ = 15 Hz is the fundamental frequency.

The third simulated signal *x*_3_(*t*) is used to simulate the noise, and its value can be generated in MATLAB using A*randn(1,*N*), where *A* = 1.5 and *N* = 2048.

The fourth simulated signal *x*_4_(*t*) can be described as: *x*_4_(*t*) = *x*_1_(*t*) + *x*_3_(*t*). The fifth simulated signal *x*_5_(*t*) was obtained by adding a single impulse to* x*_3_(*t*).

**Figure 2:**
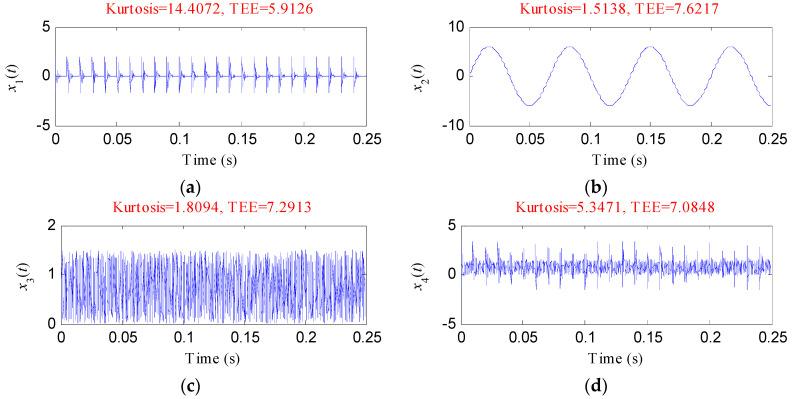

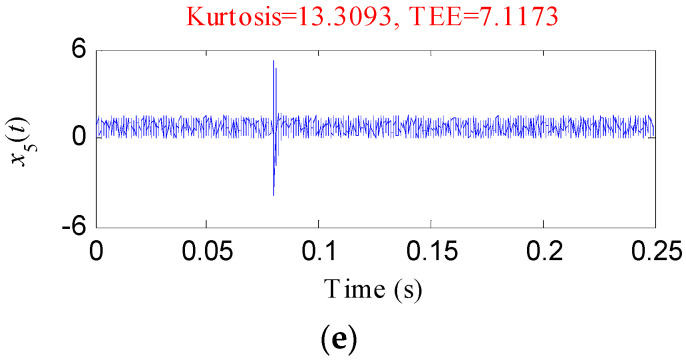
The Five simulation signal*s*: (**a**) *x*_1_(*t*); (**b**) *x*_2_(*t*); (**c**) *x*_3_(*t*); (**d**) *x*_4_(*t*); (**e**) *x*_5_(*t*).

Figure 2 depicts the waveforms of the five simulated signals and their calculated values of kurtosis and TEE. As shown in Figure 2, the kurtosis value of *x*_1_(*t*) is 14.4072, which is the highest and the TEE value of *x*_1_(*t*) is 5.9126, which is the smallest. *x*_2_(*t*) and *x*_3_(*t*) have small kurtosis values and high TEE values. Both the kurtosis and TEE indexes measure the impulsive characteristics of *x*_1_(*t*), *x*_2_(*t*) and *x*_3_(*t*) effectively. Whereas, the kurtosis value of *x*_5_(*t*) is higher than that of *x*_4_(*t*), which demonstrates that the accidental pulse results in an erroneous evaluation when using the kurtosis index. The TEE value of *x*_5_(*t*) is higher than that of *x*_4_(*t*), which demonstrates that the TEE index can be also effective when an accidental pulse appears. And it should be noticed that a smaller TEE value means that the periodic pulse characteristics of the signal are more prominent.

**Figure 3:**
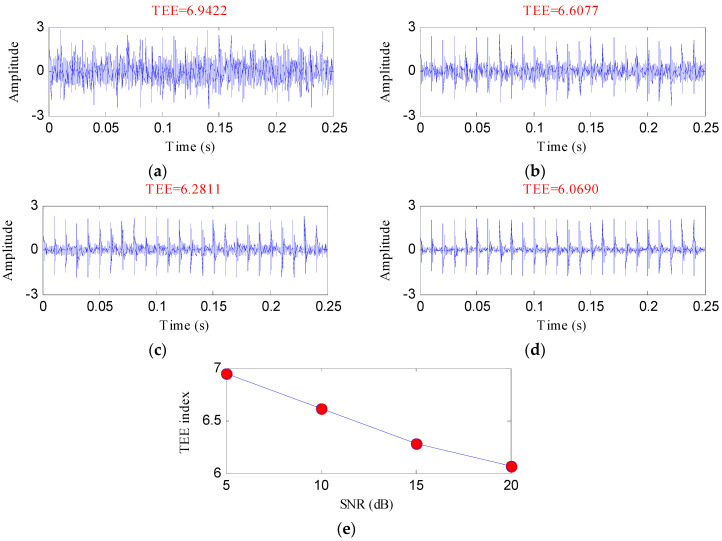
The evaluation results of the simulated signals with signal-to-noise ratios (SNRs) via Teager energy entropy (TEE) index: (**a**) 5 dB; (**b**) 10 dB; (**c**) 15 dB; (**d**) 20 dB; (**e**) the cure of the TEE index with different SNRs.

Then varying degrees of noises were added to the periodic shock feature signal of Figure 2a to obtain the signals with different signal-to-noise ratios (SNRs). The periodic fault characteristics are more obvious when the SNR of the signal is higher. By calculating the TEE values of these signals, the effectiveness of the TEE index for evaluating the degree of the periodic shock feature signal can be further verified as Figure 3 shows.

From Figure 3, it is noticeable that the value of the TEE index is smaller when the SNR is higher. This is because the impact regularity of the signal decreases due to the effect of noise. The evaluation results in this section demonstrate the TEE index is effective for evaluating the shock fault feature signals.

#### 2.1.3. Optimal Bandwidth Selection Based on Teager Energy Entropy Index

**Figure 4:**
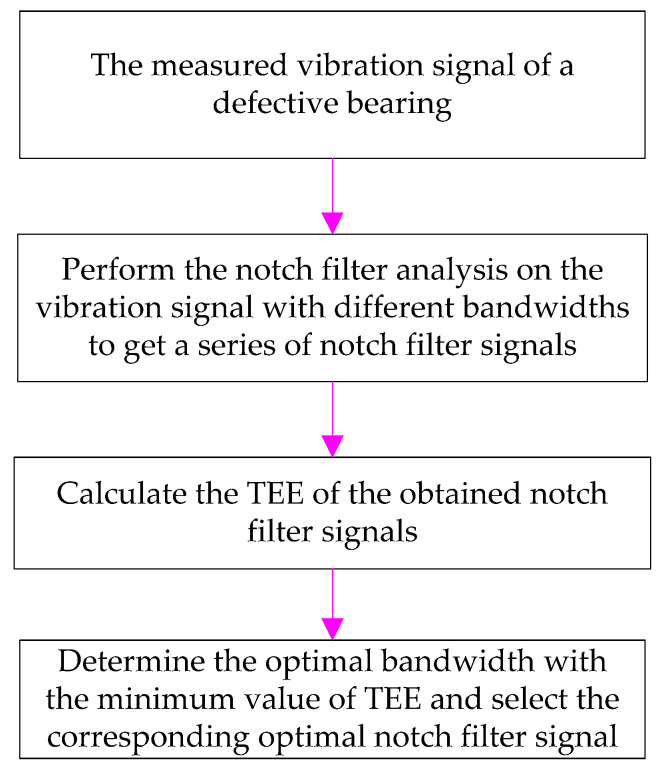
Flowchart of the optimal notch filter method.

Motivated by the above analysis, an optimal notch filter analysis method is proposed by selecting the optimal bandwidth of notch filter based on the TEE index. Figure 4 shows the flowchart of the optimal notch filter approach and the specific implementation processes are as follows:

(1) Measure the vibration signal of the defective bearing.
(2) Set the fundamental frequency as the center frequency of the notch filter and perform the notch filter analysis with varying *Bws* (*Bw *= [0.01*f_s_*, 0.99*f_s_*], the step length is 0.01*f_s_*) to achieve a series of notch filter signals.
(3) Calculate the TEE value of each notch filter signal, determine the optimal bandwidth with the smallest TEE value and select the corresponding notch filter signal as the optimal notch filter signal.

If the fundamental frequency signal plays a role as an interference signal in the raw vibration signal, the amplitudes of the fundamental frequency and its harmonics can be generally identified from the envelope spectrum of the raw vibration signal. So we can judge that whether the fundamental frequency signal is an interference signal by observing the envelope spectrum of the raw vibration signal. As reported in [18,36], the amplitude energy ration is an effective indicator to evaluate the richness of one signal component in the raw vibration signal. Inspired by the ideas in [18,36], the fundamental frequency amplitude energy ration (FFAER) indicator was introduced to automatically judge whether the fundamental frequency signal is an interference:

(8) $$FFAER=\frac{A^{2}(f_{r})+A^{2}(2f_{r})+A^{2}(3f_{r})}{W},$$

where *f_r_* is the fundamental frequency, *A*(*f_r_*), *A*(2*f_r_*), *A*(3*f_r_*) represent the amplitude of *f_r_*, 2*f_r_*, 3*f_r_* respectively in the envelope spectrum of the original signal, and *W* represents the total energy of the local envelope spectrum in the frequency band [0, 3*f_r_*].

Equation (8) is an empirical formula, and a threshold (10% was recommended in this paper) is used to evaluate whether the fundamental signal is an interference based on the results of many tests. If the value of FFAER of the signal is greater than the threshold, the original signal would be processed by the optimal notch filter to eliminate the interference of the fundamental frequency signal.

### 2.2. Enhanced Singular Value Decomposition

For a discrete signal, *s*(*i*), *i *= 1, 2, …, *N*, its Hankel matrix can be constructed as [37]:

(9) $$H={\left[\begin{array}{cccc} s(1) & s(2) & \cdots & s(n)\\ s(2) & s(3) & \cdots & s(n+1)\\ \vdots & \vdots & \vdots & \vdots \\ s(m) & s(m+1) & \cdots & s(N) \end{array}\right]}_{m\times n},$$

where 1 < *n *< *N*, *m *= *N* − *n *+ 1. The values of *n* and* m* are respectively set as *n *= *N*/2 and *m *= *N*/2 + 1 according to the selection method presented in [37].

The essence of SVD is the orthogonal decomposition of the Hankel matrix, which can be expressed as:

(10) $$H=UDV^{T},$$

where ***U*** ϵ **R***^m^*^×*m*^ and ***V*** ϵ **R***^n^*^×*n*^ are orthogonal matrices, ***D*** ϵ **R***^m^*^×*n*^ is the obtained diagonal matrix. The expressions of the three matrices are as follows:

(11) $$U=\left[u_{1},u_{2},\cdots ,u_{m}\right],$$

(12) $$V=\left[v_{1},v_{2},\cdots ,v_{n}\right],$$

(13) $$D=\left[\mathrm{diag}[\sigma_{1},\sigma_{2},\cdots ,\sigma_{q}],0\right],$$

where *σ_i_* (*i *= 1, 2, …, *q*) denotes the singular values of the matrix ***H***, *q* is the number of the nonzero singular values. With a comprehensive consideration of Equations (10)–(13), ***H*** can be described as Equation (14):

(14) $$H=\sigma_{1}u_{1}v_{1}{}^{T}+\sigma_{2}u_{2}v_{2}{}^{T}+\ldots +\sigma_{q}u_{q}v_{q}{}^{T}.$$

Let ***H****_i_* = *σ_i_**u**_i_**v**_i_*^T^ (*i *= 1, 2, …, *q*), denotes the corresponding matrix component of the singular value *σ_i_*. Therefore, the matrix ***H*** can be expressed as:

(15) $$H=H_{1}+H_{2}+\cdots +H_{q}.$$

In order to decrease the redundancy of matrix ***H***, it is necessary to select the effective singular values and corresponding matrix components to reconstruct the matrix. A series of criterions have been proposed by scholars to select the proper singular components [38], and the difference spectrum of singular values (DSSV) [37] is the most widely used one. The number of the effective singular values can be determined by observing the difference spectrum sequence. The DSSV is achieved by backward reduction of singular values:

(16) $$b_{i}=\sigma_{i}-\sigma_{i+1},\;i=1,2,\cdots ,q-1.$$

All *b_i_* sets {*b*_1_,* b*_2_, …, *b_q_*_−1_}, is called the DSSV. As pointed out in [38], the maximum peak point *b_k _*is the cut-off point of effective and useless singular values. Then the matrix ***H*** is reconstructed using the former *k* singular value components:

(17) $$H^{\prime }=H_{1}+H_{2}+\cdots +H_{k},$$

where $H^{\prime }$ is the reconstructed matrix, and the de-noised signal *y*(*i*), *i *= 1, 2, …, *N*, could be generated by the diagonal mean of $H^{\prime }$.

Though the SNR of the vibration signal can be increased by using the selection criterion of DSSV, the impact features may be lost by reconstructing the de-noised signal only using the former *k* singular values based on DSSV. To avoid missing the useful information, this work adopts the method proposed in [39], which determines the number of the effective singular values according to the peak value group of DSSV and selects the last relative maximum peak point as the demarcation point of the useful components and noise to conduct the SVD de-noising method. In order to identify the last maximum peak of DSSV adaptively, the definition of relative amplitude ration (RAR) of DSSV was given:

(18) $$RAR(i)=\frac{b_{i}}{\max(b_{i})},\;i=1,2,\cdots ,q-1,$$

where *RAR*(*i*) represents the RAR of *b_i_*. If *RAR*(*j*) is smaller than a threshold, for *j *> *m*, *j *= *m* + 1, *m* + 2, …, *q* − 1, the last maximum peak of DSSV can be identified as *b_m_*. When the threshold is set to a smaller value, more singular values are selected for reconstruction, that means more information and more noise of the raw signal will be retained. On the contrary, when the threshold is set to a greater value, the reconstructed signal will contains less noise and some useful information may be also lost. The threshold was set to 10% in this paper based on many tests.

To further eliminate the remaining noise of the SVD de-noised signal *y*(*i*), *i* = 1, 2, …, *N*, the SVD de-noised signal is multiplied a weight factor of amplitude to obtain the ESVD de-noised signal as follows:

(19) $$h(i)=y(i)\times {\left[\frac{y(i)}{p}\right]}^{2},\;i=1,2,\ldots ,N,$$

where *p* is the maximum of *y*(*i*), *i *= 1, 2, …, *N*, and *h*(*i*), *i *= 1, 2, …, *N*, represents the ESVD de-noised signal. The weight factor ${\left[\frac{y(i)}{p}\right]}^{2}$ (*i *= 1, 2, …, *N*) is a positive number less than 1. Because the amplitudes of the noise interferences are relatively smaller than the fault feature signal carried by the SVD de-noised signal, the noise interferences can be suppressed further by multiplying the weight factor, and the main components can become more prominent.

### 2.3. The Presented Method

**Figure 5:**
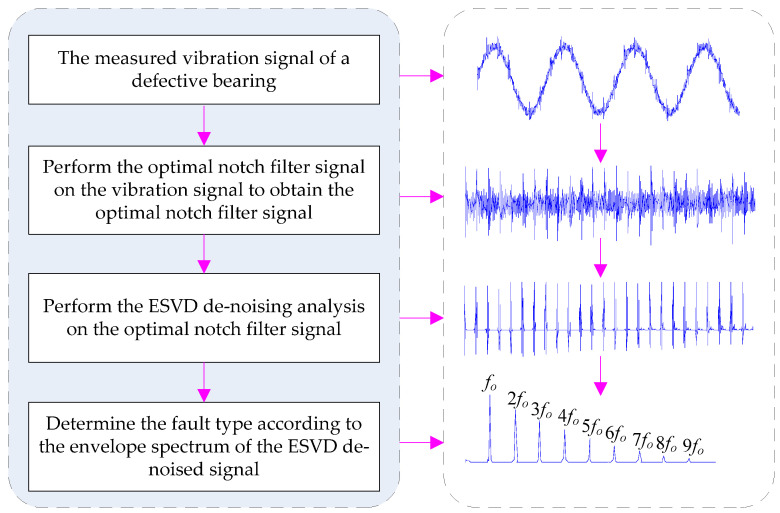
Framework of the presented method.

To suppress the compound interference of the fundamental frequency signal and background noise, the optimal notch filter was combined with the ESVD de-noising for bearing failure detection. First, the raw vibration signal was analyzed by optimal notch filter analysis to get the optimal notch filter signal. Then, the ESVD de-noising analysis was conducted on the optimal notch filter signal to get the ESVD de-noised signal. Finally the de-noised signal was analyzed by envelope spectrum analysis to obtain the fault features. Figure 5 describes the principle of the proposed method.

## 3. Simulated Analysis

To investigate the proposed method, a multi-component signal constructed based on Equation (20) is used for analysis in this section.

(20) $$S(t)=x_{1}(t)+x_{2}(t)+x_{3}(t).$$

As mentioned in Section 2.1.2, *x*_1_(*t*) represents the simulated outer race (OR) fault signal and the OR characteristic frequency *f_o_* = 1/*T_o_*= 100 Hz. *x*_2_(*t*) and *x*_3_(*t*) are used for simulating the fundamental frequency signal and noise, respectively. The simulated OR fault signal, fundamental frequency signal and noise component were respectively shown in Figure 2a–c in above analysis. A sampling frequency of 8192 Hz was used and the signal length of *S*(*t*) is 2048.

**Figure 6:**
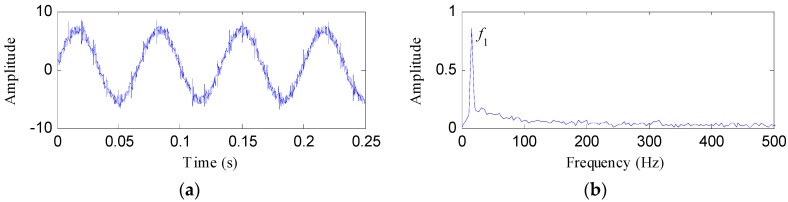
The simulated signal *S*(*t*): (**a**) its time waveform; (**b**) its envelope spectrum.

**Figure 7:**
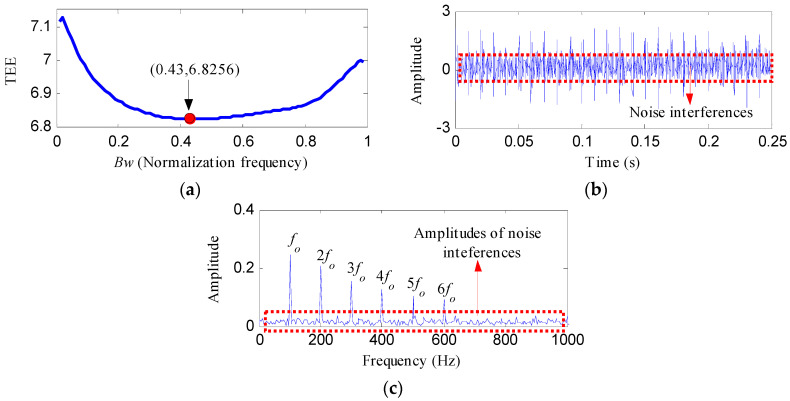
The analysis results of *S*(*t*) using the optimal notch filter analysis: (**a**) the curve of the TEE index under different bandwidths; (**b**) the optimal notch filter signal; (**c**) the envelope spectrum of (**b**).

Figure 6 depicts the waveform in time domain and envelope spectrum of *S*(*t*). It is hard to identify the impulsive features of OR fault from the waveform. The value of the FFAER indicator of the simulated signal is 73.18%, which indicates that the fundamental frequency signal performs very prominent in the simulated signal. Meanwhile, only the frequency of *x*_2_(*t*), that is *f*_1_, could be found from the envelope spectrum. Then, the simulated signal was processed by notch filters with *f*_1 _as the center frequency, and with varying *Bw*s (from 0.01*f_s_* to 0.99*f_s_* and the step length was 0.01*f_s_*). The TEE values of the notch filter signals under different *Bw*s were exhibited in Figure 7a. From Figure 7a, it can be found that the TEE value is minimum when *Bw* = 0.43*f_s_*. So the optimal *Bw *is 0.43*f_s_* and the corresponding optimal notch filter signal was depicted in Figure 7b. The optimal notch filter signal reflects the same impulsive feature with *x*_1_(*t*). However, the interferences are very obvious. Figure 7c shows the envelope spectrum of the optimal notch filter signal, some apparent peaks can be visible at the frequencies of *f_o_*, 2*f_o_*, 3*f_o_*, 4*f_o_*, 5*f_o_* and 6*f_o_*, but the noise interferences are also visible.

**Figure 8:**
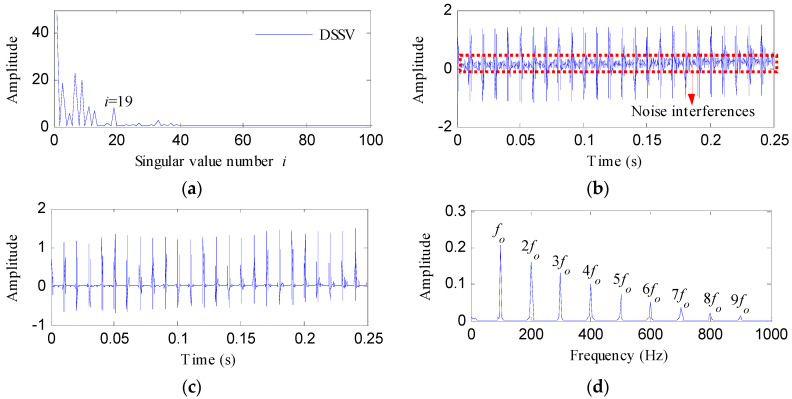
The ESVD de-noising results of the optimal notch signal: (**a**) the DSSV; (**b**) the SVD de-noised signal; (**c**) the ESVD de-noised signal; (**d**) the envelope spectrum of (**c**).

To decrease the influence of the noise interference on the optimal notch filter signal, the ESVD de-noising analysis was performed on the optimal notch filter signal. Figure 8a shows the DSSV obtained by conducting SVD on the optimal notch filter signal, from which we can see that the last relatively apparent maximum peak point appears when the serial number of the singular value is 19. Therefore, the first 19 singular components were adopted to construct the SVD de-noised signal as shown in Figure 8b. Figure 8c displays the ESVD de-noised signal. The ESVD de-noised signal reflects more prominent impact features compared with the SVD de-noised signal and the optimal notch filter signal. Consequently, the envelope spectrum of the ESVD de-noised signal shown in Figure 8d reflects more harmonics than the envelope spectrum of the optimal notch filter signal, meanwhile, no amplitudes of noise could be found from Figure 8d. The simulation signal analysis results reflect the validity of the presented method in overcoming the compound interference of noise and fundamental frequency signal, and enhancing the impulsive feature signal.

**Figure 9:**
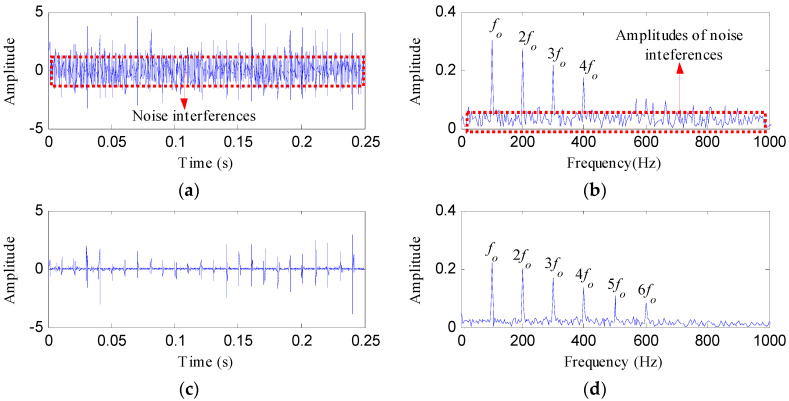
The analysis results of *S*(*t*) using MED: (**a**) the filtered signal; (**b**) its envelope spectrum; (**c**) the ESVD de-noised signal of (**a**); (**d**) the envelope spectrum of (**c**).

**Figure 10:**
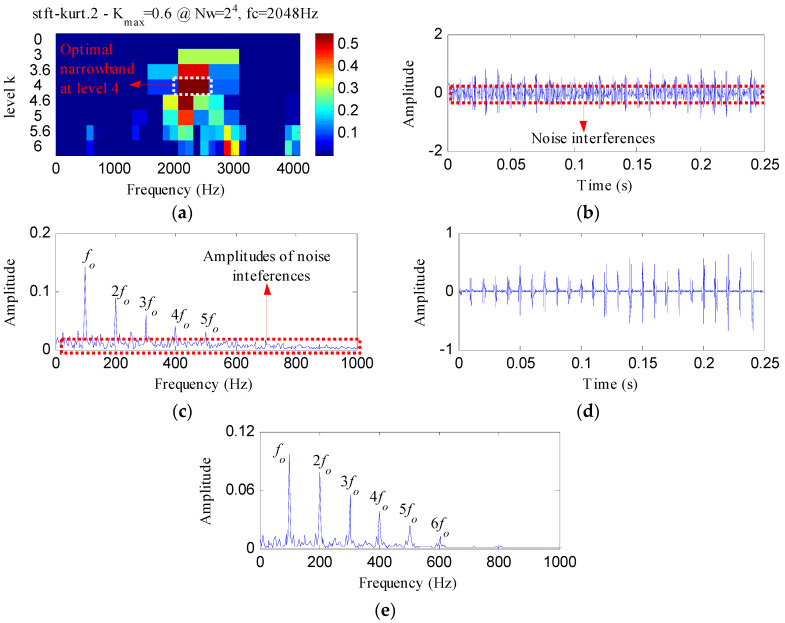
The analysis results of *S*(*t*) using Kurtogram: (**a**) the Kurtogram; (**b**) the filtered signal of the optimal narrowband; (**c**) the envelope spectrum of (**b**); (**d**) the ESVD de-noised signal of (**b**); (**e**) the envelope spectrum of (**d**).

**Figure 11:**
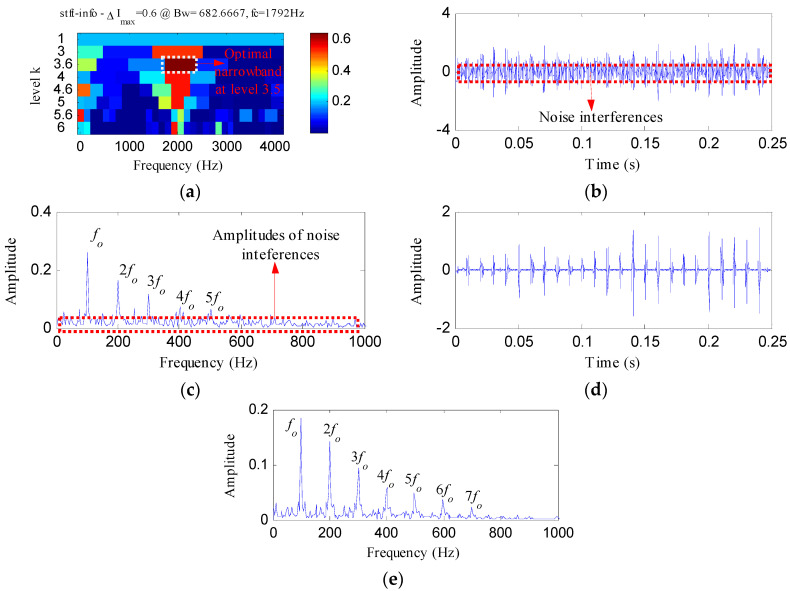
The analysis results of *S*(*t*) using Infogram: (**a**) the average infogram ∆***I***_1/2_(***f***; ∆***f***); (**b**) the filtered signal of the optimal narrowband; (**c**) the envelope spectrum of (**b**); (**d**) the ESVD de-noised signal of (**b**); (**e**) the envelope spectrum of (**d**).

The proposed method was compared with three methods, i.e., minimum entropy deconvolution (MED), Kurtogram and Infogram, to further validate its advantages. For an equitable comparison, the ESVD de-noising was also conducted on the filtered signals obtained using the three methods. Figure 9a,b respectively display the waveform and envelope spectrum of the filtered signal obtained by applying MED to *S*(*t*). From Figure 9a, a few impulse shocks can be detected, but the noise interferences are very obvious. The first four harmonics of *f_o_* and some amplitudes of noise interferences can be found from Figure 9b. Figure 9c shows the ESVD de-noised signal of Figure 9a,d displays its envelope spectrum. Some peaks can be found at the frequencies of *f_o_* and its harmonics, but less harmonics can be detected from Figure 9d compared with Figure 8d. Figure 10a displays the Kurtogram of *S*(*t*), the optimal narrowband can be identified at level 4 with center frequency of 2048 Hz. A designed band-pass filter based on the information of the optimal narrowband was conducted on the raw vibration signal and the filtered signal is plotted in Figure 10b. The filtered signal is also contaminated by noise. Figure 10c shows envelope spectrum of Figure 10b. From Figure 10c, the first five harmonics of *f_o_* and some amplitudes of noise interference can be visible. Figure 10d depicts the ESVD de-noised signal of Figure 10b. Figure 10e shows the envelope spectrum of Figure 10d, which displays less harmonics of *f_o_* compared with Figure 8d. Figure 11 reflects the analysis results obtained using the Infogram method. We can find that the proposed method shows a better performance than the Infogram method in enhancing the impulsive features and extracting more harmonics of *f_o_*.

From the simulated analysis, we can find that the optimal notch filter analysis can eliminate the interference of the fundamental frequency signal directly and effectively. The ESVD de-noising shows a better performance than the SVD de-noising in inhibiting the noise buried in the optimal notch filter signal.

## 4. Experimental Analysis

### 4.1. Experiment 1

**Figure 12:**
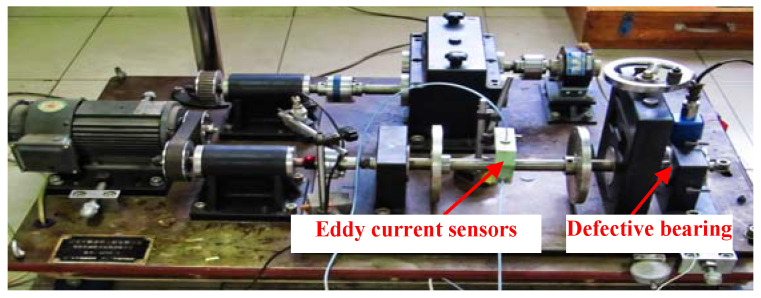
Test stand of experiment 1.

**Figure 13:**
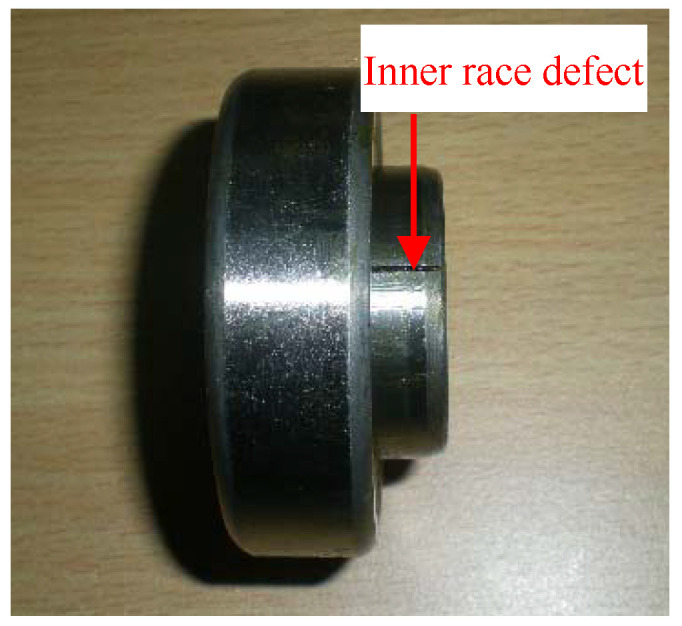
Bearing inner race defect.

Experiment 1 is conducted in the vibration test laboratory of North China Electric Power University (NCEPU). The test stand of experiment 1 is shown in Figure 12. Figure 13 displays the artificial inner race (IR) fault on the defective bearing. Table 1 gives the specific parameters of the faulty bearing. During the test, the rotating frequency (*f_r_*) was kept at 24 Hz. The vibration signals were collected by eddy current sensors as Figure 12 reflects. The sampling frequency is 12,800 Hz and the number of sampling points is 6400. The theoretical characteristic frequency of IR fault (*f_i_*) is 172 Hz.

**Figure 14:**
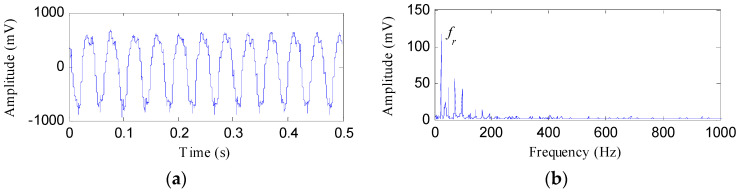
The inner race fault signal: (**a**) its time waveform; (**b**) its envelope spectrum.

The experimental IR fault signal and its envelope spectrum are shown in Figure 14. From Figure 14, we can find that the signal collected by the eddy current sensor is similar to a sinusoidal signal with burr. The FFAER value of the IR fault signal is 90.38% and the main components of the envelope spectrum are the fundamental frequency (24 Hz) and its harmonics. Whereas, the fault features of the rolling bearing could not be found from the waveform and envelope spectrum of the experimental fault signal.

**Figure 15:**
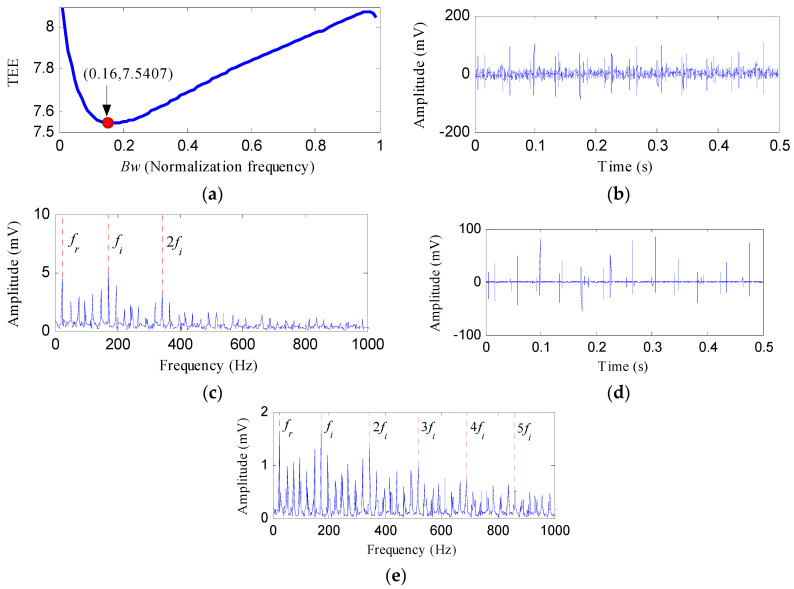
The analysis results of inner race fault signal using the proposed method: (**a**) the curve of the TEE index under different bandwidths; (**b**) the optimal notch filter signal; (**c**) the envelope spectrum of (**b**); (**d**) the ESVD de-noised signal of (**b**); (**e**) the envelope spectrum of (**d**).

The IR fault signal was processed by the proposed method and Figure 15 displays the results. By setting the center frequency as 24 Hz, and setting the *Bw* as varying values, we performed the notch filter analysis on the IR fault signal. The TEE values of the output notch filter signals under different bandwidths are shown in Figure 15a. The optimal bandwidth is 0.16*f_s_* based on the results of Figure 15a. Figure 15b shows the corresponding optimal notch signal. Prominent impact features can be detected in Figure 15b. However, the noise is also visible. From the envelope spectrum of optimal notch filter signal shown in Figure 15c, there are two peak points at the frequencies of 172 Hz and 344 Hz, corresponding to *f_i_* and 2*f_i_*, respectively. Figure 15d reflects the signal obtained by performing ESVD de-noising on the optimal notch filter signal. Compared with the optimal notch filter signal, Figure 15d presents more obvious and cleaner impact features. Figure 15e illustrates the envelope spectrum of Figure 15d. Obvious spectral lines can be detected at the frequencies of *f_i_*, 2*f_i_*, 3*f_i_*, 4*f_i_* and 5*f_i_*. Moreover, the sidebands with the interval of *f_r_* are also very clear. After the ESVD de-nosing analysis, the noise interferences of the optimal notch signal was suppressed and more harmonic frequencies of can be detected.

**Figure 16:**
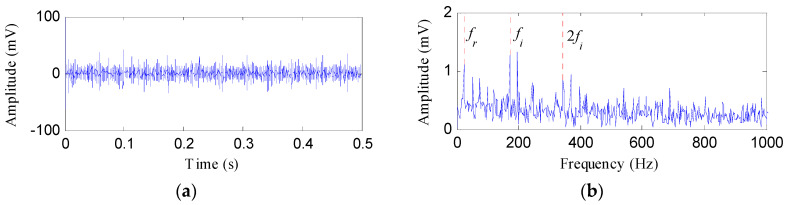

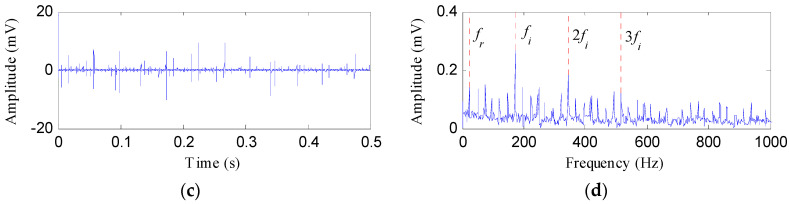
The analysis results of inner race fault signal using MED: (**a**) the filtered signal; (**b**) the envelope spectrum of (**a**); (**c**) the ESVD de-noised signal of (**a**); (**d**) the envelope spectrum of (**c**).

**Figure 17:**
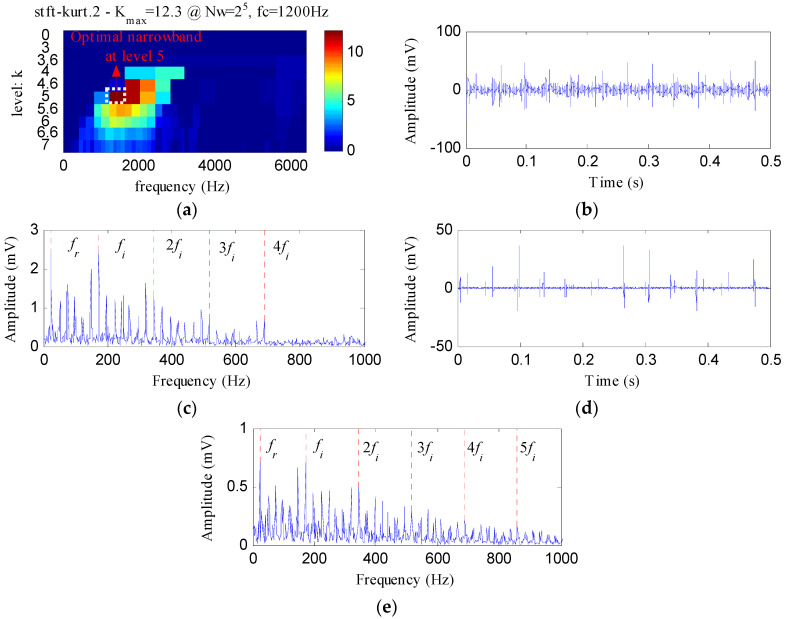
The analysis results of inner race fault signal using Kurtogram: (**a**) the Kurtogram; (**b**) the filtered signal of the optimal narrowband; (**c**) the envelope spectrum of (**b**); (**d**) the ESVD de-noised signal of (**b**); (**e**) the envelope spectrum of (**d**).

**Figure 18:**
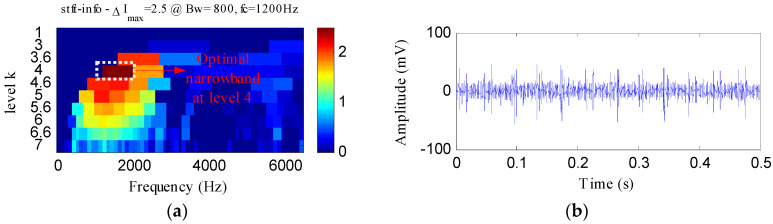

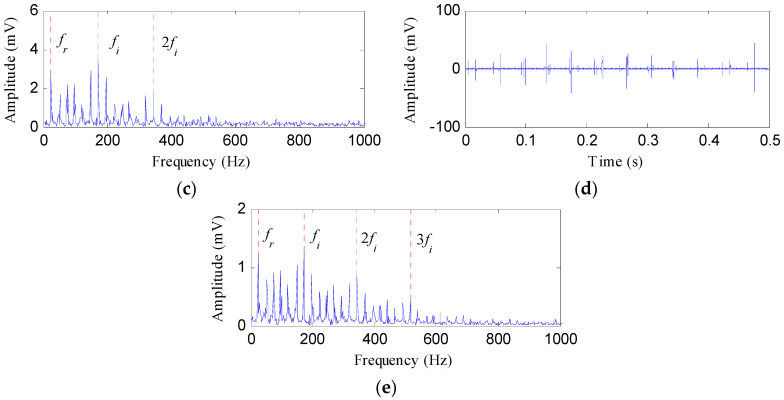
The analysis results of inner race fault signal using Infogram: (**a**) the average infogram ∆***I***_1/2_(***f***; ∆***f***); (**b**) the filtered signal of the optimal narrowband; (**c**) the envelope spectrum of (**b**); (**d**) the ESVD de-noised signal of (**b**); (**e**) the envelope spectrum of (**d**).

The IR fault signal was further analyzed by MED, Kurtogram and Infogram for comparisons. The MED filtered signal is plotted in Figure 16a,b shows its envelope spectrum. Obvious noise interferences can be visible from Figure 16a. Although, the frequencies of *f_i_* and 2*f_i_* can be detected from Figure 16b, some peaks that reflect the noise can also be visible. Figure 16c displays the signal obtained by performing ESVD de-noising on the MED filtered signal and Figure 16d shows its envelope spectrum. Only the first three harmonics of *f_i_* can be detected from Figure 16d. Figure 17a,b respectively illustrate the Kurtogram and the filtered signal obtained based on Figure 17a. Figure 17c shows the envelope spectrum of Figure 17b, which exhibits the first four harmonic frequencies of *f_i_*. Figure 17d displays the ESVD de-noised signal of Figure 17b,e displays its envelope spectrum. The first five harmonics of *f_i_* can be identified in Figure 17e. But the fourth and fifth harmonics of *f_i_* and their sidebands shown in Figure 17e are less prominent than that shown in Figure 15d obtained using the proposed method. Figure 18 shows the process results obtained via the Infogram method. By comparing Figure 18 and Figure 15, we can find that the Infogram method is not as effective as the proposed method. The comparison results demonstrate the advantages of the proposed method.

### 4.2. Experiment 2

**Figure 19:**
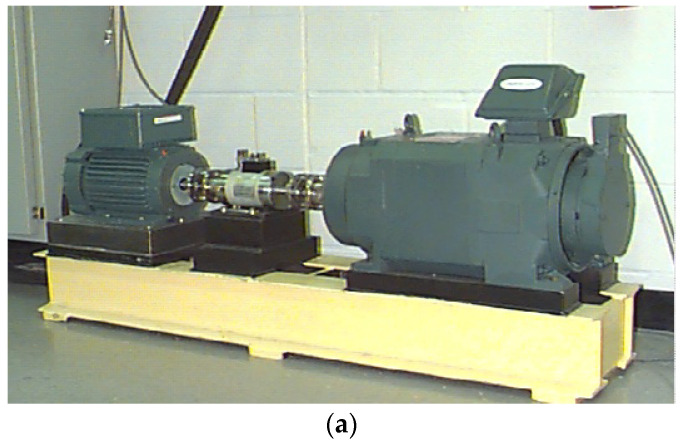

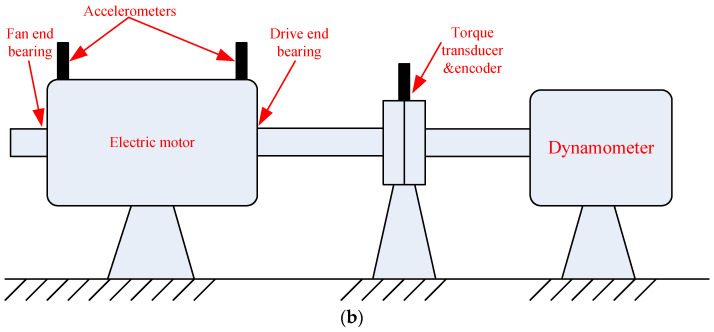
(**a**) Test stand of experiment 2; (**b**) its structure diagram.

To further investigate the presented method, the data from Case Western Reserve University (CWRU) bearing data center [40] are studied. The test stand in experiment 2 and its structure diagram are shown in Figure 19. An electric motor, a torque transducer, and a dynamometer constitute the test stand. The samples under consideration cover rolling element fault and outer race fault of the fan end bearing, the bearing type of which is SKF6203 deep groove ball. The detailed parameters of the faulty bearing are shown in Table 2. The vibration data was collected with a sampling rate of 12,000 Hz. The size defect of the studied bearing is 0.007 inches.

#### 4.2.1. Case 1: Detection of Rolling Element Defect

The considered rolling element fault sample is B007_3 X285_FE_time. To improve the efficiency of calculation, we excerpted 8192 points from the 20,000th point to the 28,191th point in the raw vibration signal. The vibration data was collected with the motor speed of 1730 rpm. The fault characteristic frequency of rolling element (*f_b_*) is about 114.97 Hz.

**Figure 20:**
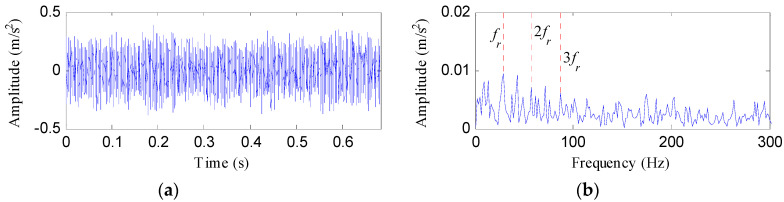
The rolling element fault signal: (**a**) its time waveform; (**b**) its envelope spectrum.

**Figure 21:**
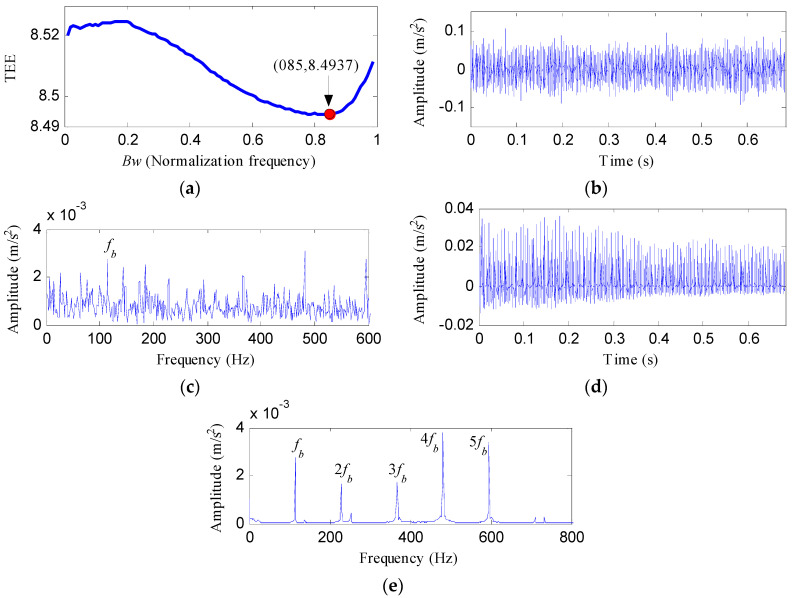
The analysis results of rolling element fault signal using the proposed method: (**a**) the curve of the TEE index under different bandwidths; (**b**) the optimal notch filter signal; (**c**) the envelope spectrum of (**b**); (**d**) the ESVD de-noised signal of (**b**); (**e**) the envelope spectrum of (**d**).

Figure 20 displays the rolling element fault signal and its envelope spectrum. The FFAER value of the rolling element fault signal was calculated as 16.6%, and only the rotating frequency (*f_r_*) and its harmonics can be detected in the envelope spectrum. It is suggested that the fundament signal plays a role as an interference signal. Then the raw vibration data was analyzed by the proposed method and the analysis results are shown in Figure 21. As Figure 21a reflects, the TEE index has the minimum value when *Bw *= 0.85*f_s_*, so we set the optimal bandwidth as 0.85*f_s_*. Figure 21b shows the waveform of the optimal notch filter signal corresponding to the optimal bandwidth, and its envelope spectrum is plotted in Figure 21c. From Figure 21c, the fault characteristic frequency of rolling element *f_b_* can be visible, but we can find some interference peaks which make it hard to identify *f_b_*. Figure 21d displays the signal obtained by performing the ESVD de-noising on the optimal notch filter signal. From Figure 21d, obvious shock features can be visible. And from the envelope spectrum of the ESVD de-noised signal, the spectral lines corresponding to the frequencies of *f_b_*, 2*f_b_*, 3*f_b_*, 4*f_b_* and 5*f_b _*are apparent. Moreover, no interferences can be visible from Figure 21e.

**Figure 22:**
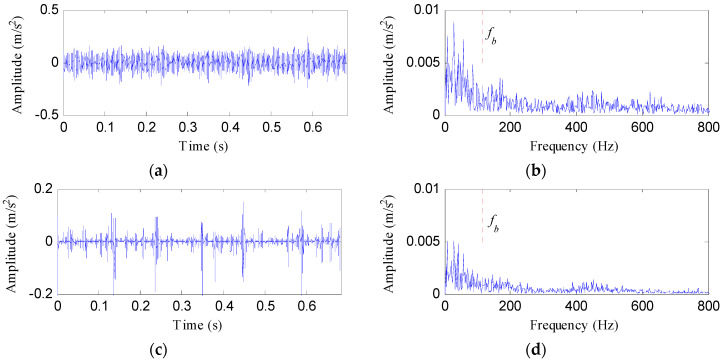
The analysis results of rolling element fault signal using MED: (**a**) the filtered signal; (**b**) the envelope spectrum of (**a**); (**c**) the ESVD de-noised signal of (**a**); (**d**) the envelope spectrum of (**c**).

**Figure 23:**
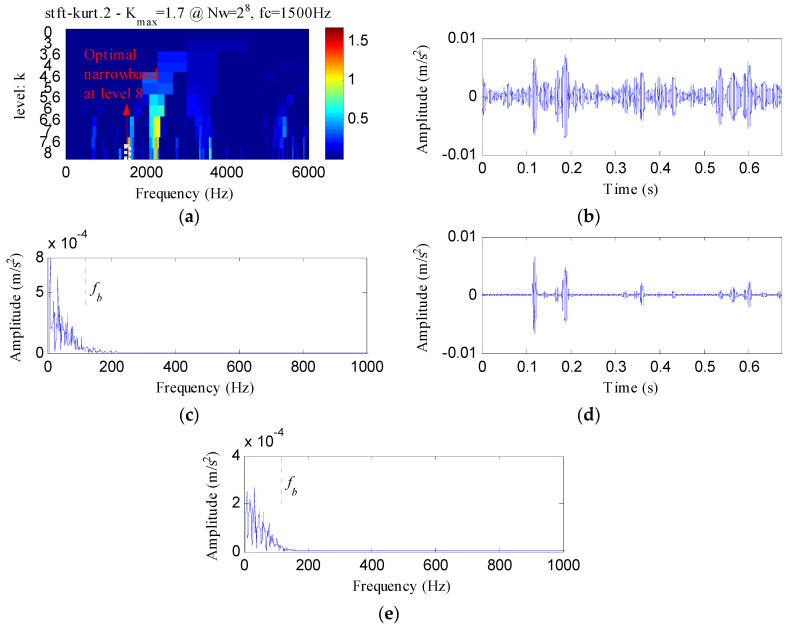
The analysis results of rolling element fault signal using Kurtogram: (**a**) the Kurtogram; (**b**) the filtered signal of the optimal narrowband; (**c**) the envelope spectrum of (**b**); (**d**) the ESVD de-noised signal of (**b**); (**e**) the envelope spectrum of (**d**).

**Figure 24:**
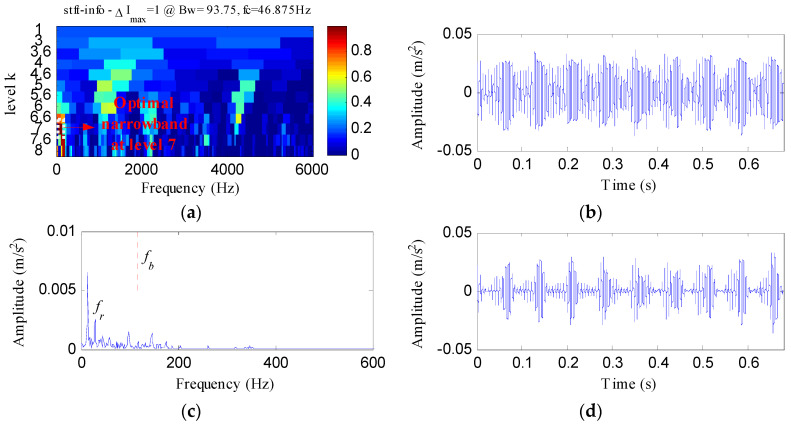

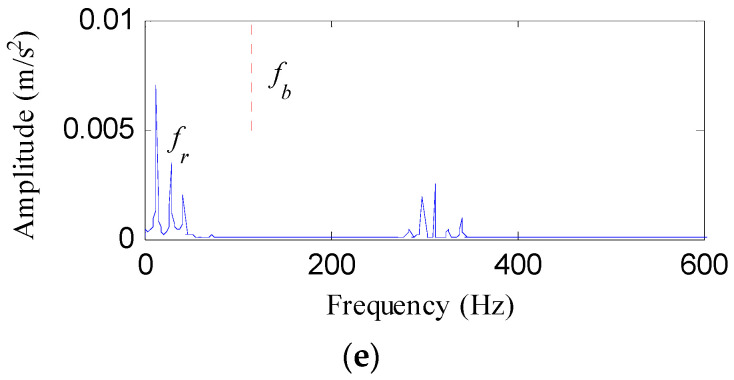
The analysis results of rolling element fault signal using Infogram: (**a**) the average infogram ∆***I***_1/2_(***f***; ∆***f***); (**b**) the filtered signal of the optimal narrowband; (**c**) the envelope spectrum of (**b**); (**d**) the ESVD de-noised signal of (**b**); (**e**) the envelope spectrum of (**d**).

The processing results of the raw vibration obtained using MED, Kurtogram and Infogram are respectively displayed in Figure 22, Figure 23 and Figure 24. None of the three methods detected the characteristic frequency of rolling element fault.

#### 4.2.2. Case 2: Detection of Outer Race Defect

The selected outer race (OR) fault sample is OR007@12_1 X305_FE_time. We excerpted the 40,000th point to the 48,191th point of the original data to improve the efficiency of calculation. The motor speed is 1772 rpm. We can calculate the fault characteristic frequency of outer race (*f_o_*) as 90.17 Hz.

**Figure 25:**
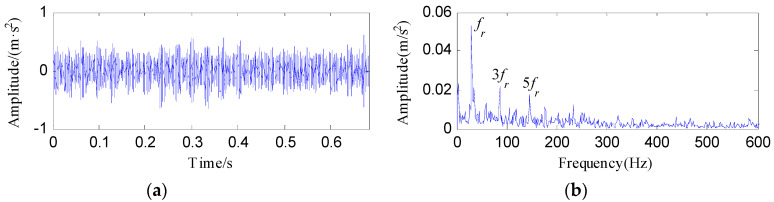
The outer race fault signal: (**a**) its time waveform; (**b**) its envelope spectrum.

Figure 25 shows the time waveform and envelope spectrum of the raw vibration signal. From the envelope spectrum, the rotating frequency and its third and fifth harmonics can be identified, but the fault characteristic frequency is invisible. The FFAER value of the raw vibration signal is 43.84%, which also reflects that the fundamental frequency signal performs very prominent.

**Figure 26:**
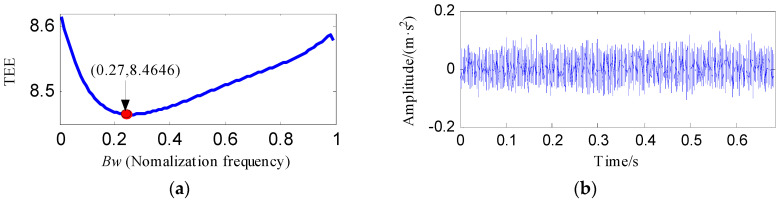

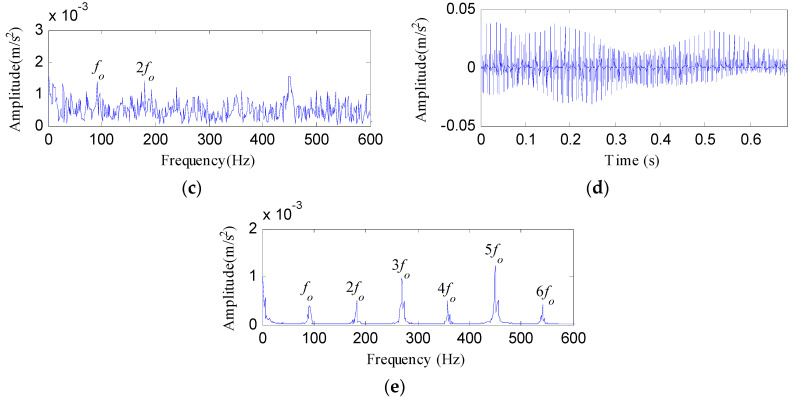
The analysis results of outer race fault signal using the proposed method: (**a**) the curve of the TEE index under different bandwidths; (**b**) the optimal notch filter signal; (**c**) the envelope spectrum of (**b**); (**d**) the ESVD de-noised signal of (**b**); (**e**) the envelope spectrum of (**d**).

The raw vibration signal was analyzed by the proposed method and Figure 26 shows the results. We can determine that the optimal bandwidth of notch filter for the OR fault signal is 0.27*f_s_* based on Figure 26a. Figure 26b displays the optimal notch filter signal corresponding to the optimal bandwidth and Figure 26c shows its envelope spectrum. Two characteristic frequencies, *f_o_* and 2*f_o_*, can be detected from Figure 26c, whereas the noise interferences are very obvious. Figure 26d reflects the de-nosed signal by performing ESVD on the optimal notch filter signal and Figure 26e shows its envelope spectrum. Some prominent peaks corresponding to the frequencies of *f_o_*, 2*f_o_*, 3*f_o_*, 4*f_o_*, 5*f_o_* and 6*f_o_* can be visible from Figure 26e. The proposed method detects the OR fault effectively.

**Figure 27:**
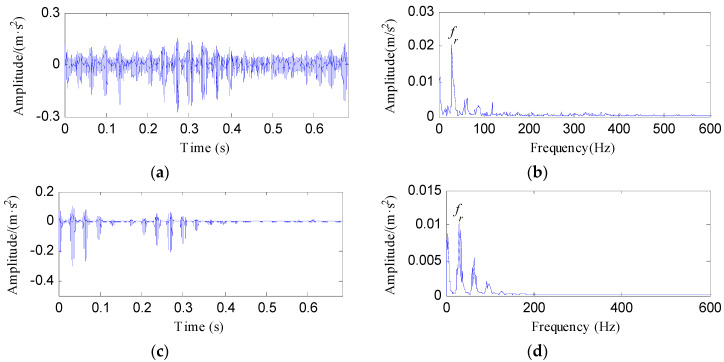
The analysis results of outer race fault signal using MED: (**a**) the filtered signal; (**b**) the envelope spectrum of (**a**); (**c**) the ESVD de-noised signal of (**a**); (**d**) the envelope spectrum of (**c**).

**Figure 28:**
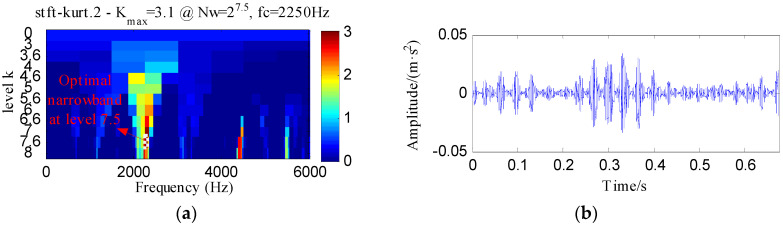

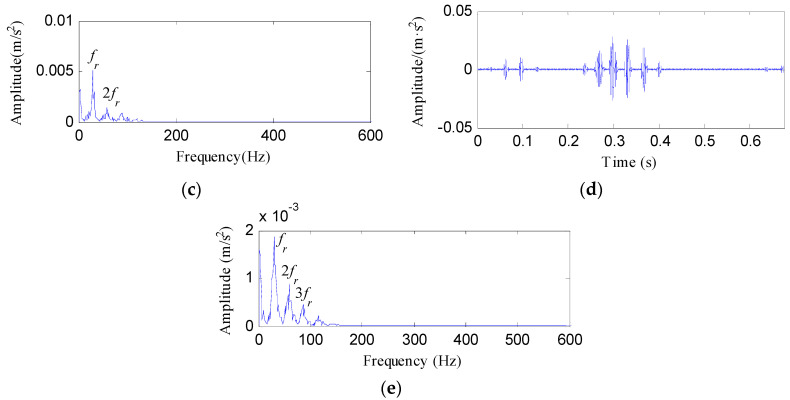
The analysis results of outer race fault signal using Kurtogarm: (**a**) the Kurtogram; (**b**) the filtered signal of the optimal narrowband; (**c**) the envelope spectrum of (**b**); (**d**) the ESVD de-noised signal of (**b**); (**e**) the envelope spectrum of (**d**).

**Figure 29:**
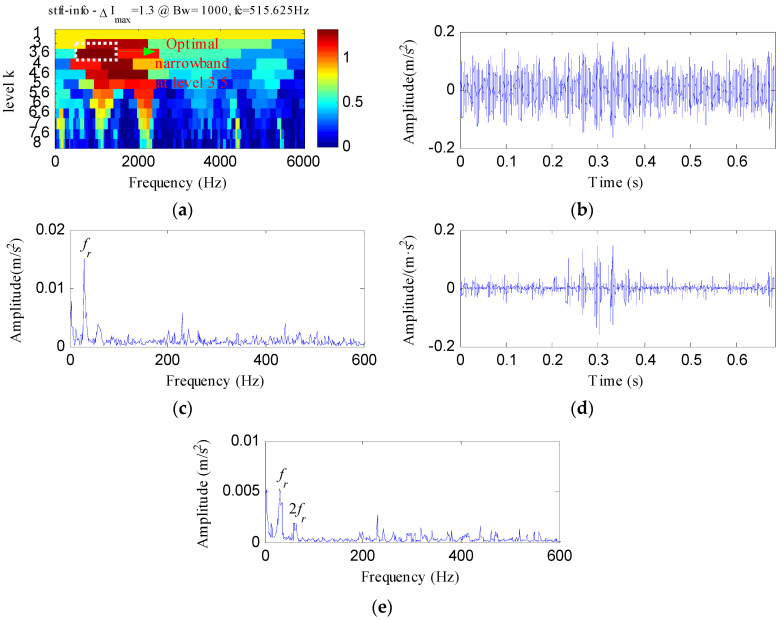
The analysis results of outer race fault signal using Infogram: (**a**) the average infogram ∆***I***_1/2_(***f***; ∆***f***); (**b**) the filtered signal of the optimal narrowband; (**c**) the envelope spectrum of (**b**); (**d**) the ESVD de-noised signal of (**b**); (**e**) the envelope spectrum of (**d**).

The OR fault signal was also analyzed by the MED, Kurtogram and Infogram methods for comparisons. Figure 27, Figure 28 and Figure 29 respectively show the analysis results. It can be found that the three methods failed to diagnose the OR fault.

## 5. Conclusions

The fundamental frequency signal is one of the most common harmonic interference signals, and the background noise can increase the difficulty of bearing fault diagnosis. To suppress the compound interference of the fundamental frequency signal and background noise, this paper proposes a novel bearing failure detection method by combing the optimal notch filter based on Teager energy entropy index and enhanced singular value decomposition de-noising. The major contributions of this work can be summarized as follows:

(1) To adaptively determine the optimal bandwidth of the notch filter and implement the optimal notch filter analysis, a new indicator for evaluating the periodic impulsive features named Teager energy entropy index was presented. The Teager energy entropy index performs better in overcoming the accidental shocks than the kurtosis index.
(2) The optimal notch filter analysis based on Teager energy entropy index (with the fundamental frequency as the center frequency) shows its ability in inhibiting the interference of the fundamental frequency signal and enhancing the shock feature signal.
(3) An enhanced singular value decomposition de-noising method was proposed to improve the noise reduction of singular value decomposition.

Both the simulated and experimental signals validate the efficiency of the proposed method for overcoming the compound interference of the fundamental frequency and the noise components.

It should be noticed that the center frequency is another key parameter of notch filter, which is set as the fundamental frequency in this paper to eliminate the interference of the fundament frequency signal. The fault feature signal may be affected by other harmonic interference signals besides the fundamental frequency signal. In our future work, the use of the notch filter analysis in suppressing other harmonics interference caused by other parts of the machine, especially the gears, will be studied. Some intelligent optimization methods such as genetic algorithm, particle swarm optimization and ant colony algorithm can be tested to adaptively set the optimal parameters of notch filter to implement the optimal notch filter analysis. The selection of the number of the effective singular values has an effect on the analysis results of the enhanced singular value decomposition. In this paper, the number of the effective singular values was determined by identifying the last relative maximum peak of the difference spectrum of singular values. In the future, we’ll consider more reasonable methods to adaptively select the number of the effective singular values.

## Figures and Tables

**Table 1:** The details of the defective bearing of experiment 1.

Roller Diameter (mm)	Pith Diameter (mm)	Number of Rollers	Contact Angle (°)
7.5	38.5	12	0°

**Table 2:** The details of the defective bearing of experiment 2.

Ball Diameter (mm)	Pith Diameter (mm)	Number of Balls	Contact Angle (°)
6.75	28.5	8	0°

## References

[1] Glowacz A., Glowacz W., Glowacz Z., Kozik J. (2018). Early fault diagnosis of bearing and stator faults of the single-phase induction motor using acoustic signals. Measurement.

[2] Yuan R., Lv Y., Song G. (2018). Multi-Fault diagnosis of rolling bearings via adaptive projection intrinsically transformed multivariate empirical mode decomposition and high order singular value decomposition. Sensors.

[3] Adamczak S., Stepien K., Wrzochal M. (2017). Comparative study of measurement systems used to evaluate vibrations of rolling bearings. Procedia Eng..

[4] Pang B., Tang G., Tian T., Zhou C. (2018). Rolling bearing fault diagnosis based on an improved HTT transform. Sensors.

[5] Zhao M., Jia X. (2017). A novel strategy for signal denoising using reweighted SVD and its applications to weak fault feature enhancement of rotating machinery. Mech. Syst. Signal Process..

[6] Li Y., Zuo M.J., Lin J., Liu J. (2017). Fault detection method for railway wheel flat using an adaptive multiscale morphological filter. Mech. Syst. Signal Process..

[7] Lv Y., Zhu Q., Yuan R. (2015). Fault diagnosis of rolling bearing based on fast nonlocal means and envelop spectrum. Sensors.

[8] He Q., Wu E., Pan Y. (2018). Multi-scale stochastic resonance spectrogram for fault diagnosis of rolling element bearings. J. Sound Vib..

[9] Liu Z., He Z., Guo W., Tang Z. (2016). A hybrid fault diagnosis method based on second generation wavelet de-noising and local mean decomposition for rotating machinery. ISA Trans..

[10] Li J., Li M., Zhang J. (2017). Rolling bearing fault diagnosis based on time-delayed feedback monostable stochastic resonance and adaptive minimum entropy deconvolution. J. Sound Vib..

[11] Miao Y., Zhao M., Lin J., Lei Y. (2017). Application of an improved maximum correlated kurtosis deconvolution method for fault diagnosis of rolling element bearings. Mech. Syst. Signal Process..

[12] Miao Y., Zhao M., Lin J., Xu X. (2016). Sparse maximum harmonics-to-noise-ratio deconvolution for weak fault signature detection in bearings. Meas. Sci. Technol..

[13] Raj A.S. (2015). A novel application of Lucy-Richardson deconvolution: Bearing fault diagnosis. J. Vib. Control.

[14] Wang Z., Wang J., Zhao Z., Wang R. (2018). A novel method for multi-fault feature extraction of a gearbox under strong background noise. Entropy.

[15] Li G., Zhao Q. (2017). Minimum entropy deconvolution optimized sinusoidal synthesis and its application to vibration based fault detection. J. Sound Vib..

[16] Yi Z., Pan N., Guo Y. (2017). Mechanical compound faults extraction based on improved frequency domain blind deconvolution algorithm. Mech. Syst. Signal Process..

[17] Zhang X., Liang Y., Zhou J., Zang Y. (2015). A novel bearing fault diagnosis model integrated permutation entropy, ensemble empirical mode decomposition and optimized svm. Measurement.

[18] Yan X., Jia M., Xiang L. (2016). Compound fault diagnosis of rotating machinery based on OVMD and a 1.5-dimension envelope spectrum. Meas. Sci. Technol..

[19] Song Y., Zeng S., Ma J., Guo J. (2018). A fault diagnosis method for roller bearing based on empirical wavelet transform decomposition with adaptive empirical mode segmentation. Measurement.

[20] Zhang L., Wang Z., Quan L. (2018). Research on weak fault extraction method for alleviating the mode mixing of LMD. Entropy.

[21] Antoni J. (2007). Fast computation of the kurtogram for the detection of transient faults. Mech. Syst. Signal Process..

[22] Moshrefzadeh A., Fasana A. (2018). The autogram: An effective approach for selecting the optimal demodulation band in rolling element bearings diagnosis. Mech. Syst. Signal Process..

[23] Antoni J. (2016). The infogram: Entropic evidence of the signature of repetitive transients. Mech. Syst. Signal Process..

[24] Wang Y., Xiang J., Markert R., Liang M. (2016). Spectral kurtosis for fault detection, diagnosis and prognostics of rotating machines: A review with applications. Mech. Syst. Signal Process..

[25] Xu X., Qiao Z., Lei Y. (2018). Repetitive transient extraction for machinery fault diagnosis using multiscale fractional order entropy infogram. Mech. Syst. Signal Process..

[26] Mojiri M., Karimi-Ghartemani M., Bakhshai A. (2007). Time-domain signal analysis using adaptive notch filter. IEEE. Trans. Signal Process..

[27] Koshita S., Noguchi Y., Abe M., Kawamata M. (2016). Analysis of frequency estimation mse for all-pass-based adaptive iir notch filters with normalized lattice structure. Signal Process..

[28] Zhao H., Li L. (2016). Fault diagnosis of wind turbine bearing based on variational mode decomposition and Teager energy operator. IET Renew. Power Gen..

[29] Wan S., Zhang X., Dou L. (2018). Shannon Entropy of Binary Wavelet Packet Subbands and Its Application in Bearing Fault Extraction. Entropy.

[30] Zheng K., Li T., Zhang B., Zhang Y., Luo J., Zhou X. (2017). Incipient Fault Feature Extraction of Rolling Bearings Using Autocorrelation Function Impulse Harmonic to Noise Ratio Index Based SVD and Teager Energy Operator. Appl. Sci..

[31] Sun P., Liao Y., Lin J. (2017). The Shock Pulse Index and Its Application in the Fault Diagnosis of Rolling Element Bearings. Sensors.

[32] Wu T.-Y., Yu C.-L., Liu D.-C. (2016). On Multi-Scale Entropy Analysis of Order-Tracking Measurement for Bearing Fault Diagnosis under Variable Speed. Entropy.

[33] Feng Z., Ma H., Zuo M.J. (2017). Spectral negentropy based sidebands and demodulation analysis for planet bearing fault diagnosis. J. Sound Vib..

[34] Eguiraun H., Casquero O., Martinez I. (2018). The Shannon Entropy Trend of a Fish System Estimated by a Machine Vision Approach Seems to Reflect the Molar Se:Hg Ratio of Its Feed. Entropy.

[35] Cong F., Zhong W., Tong S., Tang N., Chen J. (2015). Research of singular value decomposition based on slip matrix for rolling bearing fault diagnosis. J. Sound Vib..

[36] Yan X., Jia M., Zhang W., Zhu L. (2018). Fault diagnosis of rolling element bearing using a new optimal scale morphology analysis method. ISA Trans..

[37] Zhao X., Ye B. (2011). Selection of effective singular values using difference spectrum and its application to fault diagnosis of headstock. Mech. Syst. Signal Process..

[38] Alharbi N., Hassani H. (2016). A new approach for selecting the number of the eigenvalues in singular spectrum analysis. J. Frankl. Inst..

[39] Guo Y., Wei Y., Zhou X., Fu L. (2015). Impact feature extracting method based on S transform time-frequency spectrum denoised by SVD. J. Vib. Eng..

[40] Case Western Reserve University Bearing Data Center Website. https://csegroups.case.edu/bearingdatacenter/home.

